# Correlated electron-nuclear dynamics in above-threshold multiphoton ionization of asymmetric molecule

**DOI:** 10.1038/srep42585

**Published:** 2017-02-20

**Authors:** Zhuo Wang, Min Li, Yueming Zhou, Pengfei Lan, Peixiang Lu

**Affiliations:** 1 School of Physics and Wuhan National Laboratory for Optoelectronics, Huazhong University of Science and Technology, Wuhan 430074, China; 2 Laboratory of Optical Information Technology, Wuhan Institute of Technology, Wuhan 430205, China

## Abstract

The partition of the photon energy into the subsystems of molecules determines many photon-induced chemical and physical dynamics in laser-molecule interactions. The electron-nuclear energy sharing from multiphoton ionization of molecules has been used to uncover the correlated dynamics of the electron and fragments. However, most previous studies focus on symmetric molecules. Here we study the electron-nuclear energy sharing in strong-field photoionization of HeH^2+^ by solving the one-dimensional time-dependent Schrödinger equation (TDSE). Compared with symmetric molecules, the joint electron-nuclear energy spectrum (JES) of HeH^2+^ reveals an anomalous energy shift at certain nuclear energies, while it disappears at higher and lower nuclear energies. Through tracing the time evolution of the wavepacket of bound states, we identify that this energy shift originates from the joint effect of the Stark shift, associated with the permanent dipole, and the Autler-Townes effect due to the coupling of the 2*pσ* and 2*sσ* states in strong fields. The energy shift in the JES appears at certain nuclear distances only when both Stark effect and Autler-Townes effect play important roles. We further demonstrate that the electron-nuclei energy sharing can be controlled by varying laser intensity for asymmetric molecules, providing alternative approaches to manipulate photochemical reactions for more complex molecules.

The interaction of atoms and molecules with intense infrared laser pulses has been a subject of continuous studies for more than three decades^1^,^2^,^3^,^4^,^5^,^6^,^7^,^8^,^9^,^10^,^11^,^12^. Typically, both ionization and fragmentation might occur for a molecule in an intense laser field. Lying at the heart of many fascinating phenomena, such as high harmonic generation and nonsequential double ionization, the ionization is of fundamental interest. In strong-field ionization process of molecules, the motion of the nuclei is strongly coupled to the electron motion instead of being frozen^13^,^14^. Therefore, understanding the correlated dynamics of electrons and nuclei in molecules has long been one of the most challenging topics in strong-field physics^15^,^16^,^17^,^18^,^19^,^20^,^21^,^22^,^23^,^24^.

A basic question in strong-field ionization of molecules is how the photon energy is distributed among the subsystems of the molecules, especially when the molecules absorb more photons than the minimal number required for ionization. For molecules, the correlation between the ion and electron in the multiphoton process has recently been studied both in theory for 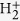
15,16,17,18,19,20,21 and in experiment for H_2_^22^ employing the JES. It is reported that electrons resulted from the multiphoton ionization will share part of their energy with the nuclei^16^,^17^. Generally, the energy sharing between the electrons and the nuclei manifests itself as many diagonal maxima separated by the laser frequency, *ω*, in the JES of the electron *E*_*e*_ and the nuclear *E*_*N*_ energies^17^ [atomic units (a.u.) are used unless stated otherwise],


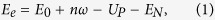


where *E*_0_ is the bound state energy, and *U*_*P*_ the ponderomotive energy, and *n* the number of absorbed photons. From equation (1), the electron energy is linear with the nuclear energy with absorption of specific numbers of photons. The slope of the lines in the JES should be −1. For symmetric 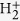 molecule, the relation of equation (1) might not be satisfied for high vibrational state because of the strong coupling between the ground state and first excited state of molecule in strong laser fields at a large nuclear distance^21^. Most recently, Yue *et al*.^19^ found more energy-sharing structures in the JES at long laser wavelength that they attributed to the intracycle interference effect. Actually, in the JES both diagonal structures with negative slope and cross-diagonal structures with positive slope can coexist. It has been demonstrated that the diagonal and cross-diagonal structures are associated with the intercycle effects and intracycle effects, respectively. It is shown that equation (1) is also satisfied for the JES of multielectron molecules CO and the JES depends strongly on the vibrational state^25^,^26^.

Most previous studies on the JES focus on symmetric molecules, such as H_2_^22^, 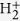
15,16,17,18,19,20,21. However, for asymmetric molecules such as HeH^2+^, some effects have been discovered associated with the permanent dipoles^27^,^28^,^29^,^30^,^31^,^32^,^33^, which are different from symmetric molecules. For example, it has been reported that for an asymmetric molecule driven by a few-cycle linearly polarized pulse, enhanced ionization is much stronger for the antiparallel orientation of the permanent dipole of the molecule relative to the electric field, than for the parallel orientation^28^,^29^,^30^. In this paper, we study the correlated electron-nuclear dynamics for asymmetric HeH^2+^ molecule. By numerically solving the TDSE, we study the electron-nuclear energy sharing in strong-field photoionization of HeH^2+^. Compared with the JES of symmetric molecules, we find an anomalous energy shift in the JES of HeH^2+^, inconsistent with the electron-nuclear energy sharing rule predicted by equation (1). This energy shift appears only at intermediate nuclear energies, whereas it disappears at higher and lower nuclear energies. By tracing the time evolution of the electron wave packet of bound states, we identify that the energy shift originates from the joint effect of the Stark shift, which is associated with the permanent dipole of HeH^2+^ molecule, and the Autler-Townes effect induced by the coupling of the 2*pσ* and 2*sσ* states of HeH^2+^ in strong laser fields. We further demonstrate that the electron-nuclei energy sharing can be controlled by varying the laser intensity for asymmetric molecules.

## Results

**Figure 1:**
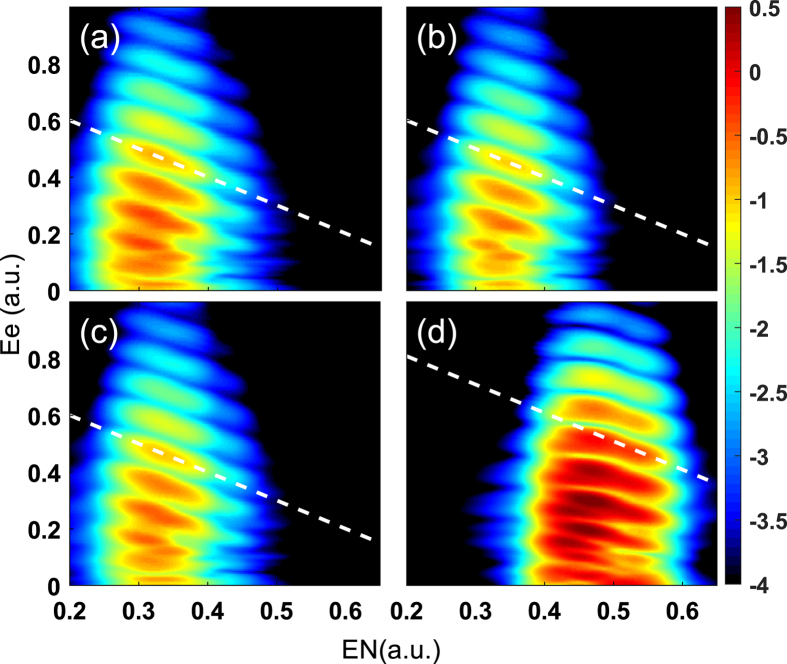
Electron-nuclear JES for the diatomic molecules. Electron-nuclear JES for the above-threshold multiphoton ionization processes of the diatomic molecules exposed to a 400 nm, 2 × 10^14^ W/cm^2^ laser pulse for (**a**) 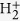, (**b**) 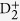, (**c**) HD^+^, and (**d**) HeH^2+^, respectively. The initial vibrational states are all *υ* = 0. The color scale is logarithmic. The white dashed lines are used to guide the electron-energy sharing in strong field ionization for different molecules with absorption of 15 photons for symmetric molecules and 17 photons for HeH^2+^ molecule. The slopes of them are −1.

We first show the electron-nuclear JES for the above-threshold multiphoton ionization of different diatomic molecules exposed to a 400 nm, 2 × 10^14^ W/cm^2^ laser pulse in Fig. 1. The JES are obtained by using the method from ref. 16 with numerically solving the one-dimensional TDSE. Figure 1(a–d) show the JES for 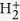, 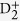, HD^+^ and HeH^2+^, respectively. The initial states of Fig. 1(a–c) are set to be the ground states of 1*sσ (υ* = 0), while the initial state of Fig. 1(d) is the first excited state (2*pσ, υ* = 0) of HeH^2+^ molecule. To guide the eyes, we show a white dashed line with slope of −1 in each subplot with absorption of a specific number of photons. Generally, for the four diatomic molecules, one can see multiple tilted stripe structures spaced by *ω*, revealing the energy sharing between the electron and nuclei with multiphoton absorption. For the asymmetric HeH^2+^ molecule [Fig. 1(d)], however, a characteristic feature is distinctly different from the case of the symmetric molecules [Fig. 1(a–c)]. For the symmetric diatomic molecule, the slopes of the tilted stripes are all −1, consisting with the electron-nuclear energy-sharing rule predicted by equation (1). However, the tilted stripes look more complex in Fig. 1(d), which is inconsistent with the prediction of equation (1). The peak positions of the JES exhibit obvious energy shifts with respect to the dashed line, as shown in Fig. 1(d). The energy shift can be more clearly seen with the decrease of the nuclear energies. It is important to emphasize that this energy shift in the JES of the asymmetric HeH^2+^ is different from that of the symmetric 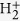
21, where no energy shift is observed for low vibrational states.

**Figure 2:**
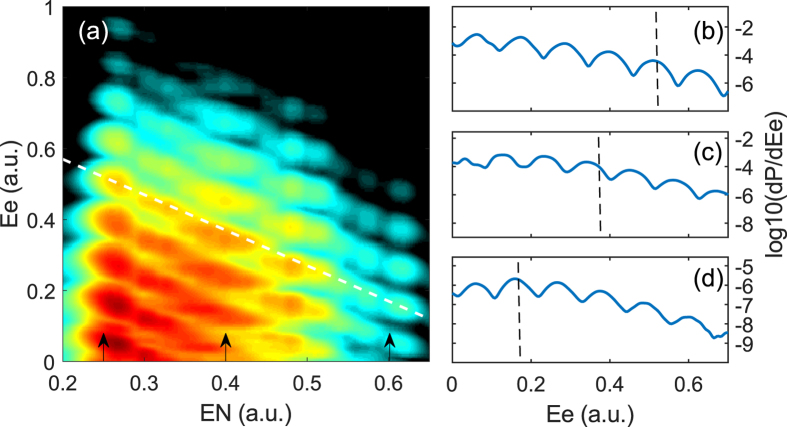
Electron-nuclear JES of HeH^2+^ for the high vibrational state. (**a**) Electron-nuclear JES for the above-threshold multiphoton ionization process of HeH^2+^ (*υ* = 17) exposed to a 400 nm, 1.4 × 10^14^ W/cm^2^ laser pulse. The color scale is logarithmic. The white dashed line, with the slope of −1, indicates the absorption of 15 photons. (**b**–**d**) are the cuts for the nuclear energies *E*_*N*_ = 0.25 a.u., *E*_*N*_ = 0.4 a.u., and *E*_*N*_ = 0.6 a.u., respectively, as indicated by the arrows in (**a**). The vertical dashed lines indicate the positions of the white dashed line in (**a**).

To see if this energy shift in the JES depends on the vibrational state we calculate the electron-nuclear JES of a high vibrational state of HeH^2+^ (*υ* = 17) exposed to a 400 nm laser pulse, as shown in Fig. 2(a). The high vibrational state allows one to see the energy shift in the JES from small to large nuclear energies. We also show the prediction of equation (1) by the white dashed line with a slope of −1 in Fig. 2(a). To see the energy shift more clearly, we show the cuts for the nuclear energies *E*_*N*_ = 0.25 a.u. [Fig. 2(b)], *E*_*N*_ = 0.4 a.u. [Fig. 2(c)], and *E*_*N*_ = 0.6 a.u. [Fig. 2(d)], respectively, as indicated by the arrows in Fig. 2(a). The vertical dashed lines indicate the positions of the white dashed line in Fig. 2(a). Interestingly, the energy shift of the JES for the asymmetric HeH^2+^ only appears at the intermediate nuclear energies (e.g. *E*_*N*_ = 0.4 a.u.) and it disappears at the lower (e.g. *E*_*N*_ = 0.25 a.u.) and higher (*E*_*N*_ = 0.6 a.u.) nuclear energies. This is very different from the energy shift for the high vibrational states of symmetric 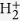 molecule, which becomes larger for lower nuclear energies^21^. Additionally, we can see the ionization probability (color scale) is enhanced at the nuclear energies *E*_*N*_ ≈ 0.42 a.u. and *E*_*N*_ ≈ 0.28 a.u. in Figs 1(d) and 2(a), respectively. This is known as the enhanced ionization for the asymmetric HeH^2+^ molecule, which occurs at some intensity-dependent critical internuclear distances^28^,^29^,^30^. The critical internuclear distances at which the enhanced ionization occurs in Figs 1(d) and 2(a) are consistent with the previous work^29^, indicating that the one-dimensional model is reliable.

**Figure 3:**
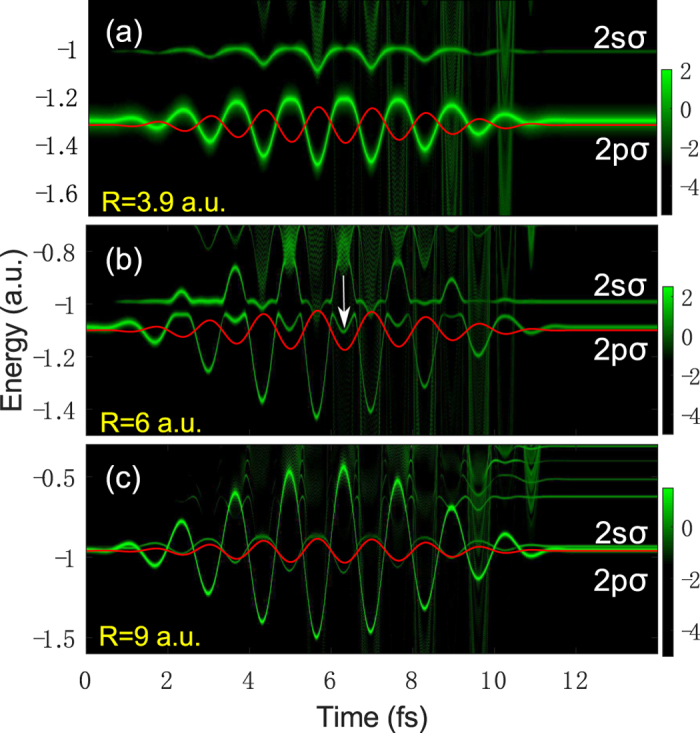
The time evolution of the electron wave packet. The time evolution of the electron wave packet of bound states of HeH^2+^ for different internuclear distances (**a**) *R* = 3.9 a.u., (**b**) *R* = 6 a.u., and (**c**) *R* = 9 a.u., corresponding the higher, intermediate, and lower nuclear energies, respectively. The color scale is logarithmic with arbitrary units. The red solid curves represent the electric fields. The laser parameters are the same as those in Fig. 1(d).

Previously, we have demonstrated that the electron-nuclear JES obtained by the frozen nuclei model is very similar to the JES obtained by the reduced-dimensionality model^21^. For simplicity, we next reveal the origin of the energy shift observed in the JES of HeH^2+^ using the frozen nuclei model. As shown in Fig. 3(a–c), we calculate the time evolution of the electron wave packet of the bound states of HeH^2+^ using the energy window operator, which is illustrated in Section 2.2, at different internuclear distances, i.e., *R* = 3.9 a.u., *R* = 6 a.u., and *R* = 9 a.u., respectively. The laser parameters are the same as those in Fig. 1(d). The red solid curves represent the electric fields. The energies of the 2*pσ* and 2*sσ* states are labeled in Fig. 3. The color indicates the population density of the bound states. From Fig. 3(a–c), one can clearly see that all the bound states energies of HeH^2+^ oscillate with the evolution of the laser field. After the laser field is turned off, the energies of the bound states become undisturbed. We focus on the 2*pσ* and 2*sσ* states. Obviously, the oscillation of the 2*pσ* state is consistent with −*F*(*t*) for the three internuclear distances, and the amplitude of the oscillation becomes larger with the increase of the internuclear distance. On the other hand, the oscillation of the 2*sσ* state is consistent with *F*(*t*) when *R* = 9 a.u., while it coincides with -*F*(*t*) when *R* = 3.9 a.u. and *R* = 6 a.u.. The amplitude of the oscillation of the 2*sσ* is smaller than that of the 2*pσ* state. The oscillation behavior of the electronic state of the asymmetric HeH^2+^ is distinctly different from that of the symmetric 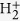, where the oscillation of the ground state and the first excited state is consistent with the shape of -|*F*(*t*)| or |*F*(*t*)|, respectively^21^. Interestingly, for HeH^2+^, at *R* = 6 a.u., we can see obvious dips (indicated by the white arrow) at the negative peaks of the laser fields for the 2*pσ* state. The internuclear distance of 6 a.u. corresponds to nuclear energy of 0.33 a.u., which has a large energy shift in the JES of Fig. 1(d). In contrast to asymmetric molecules, no dip structure can be observed in the evolution of the bound state for the symmetric molecules^21^. This dip structure can be hardly seen for smaller and larger internuclear distances, as seen in Fig. 3(a,c), respectively. As we will show below, this dip structure is associated with the energy shift in Figs 1(d) and 2(a).

**Figure 4:**
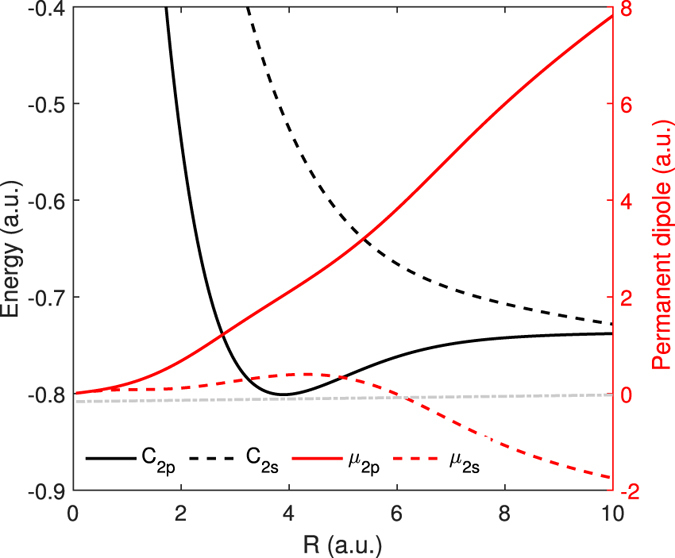
The potential curves and the permanent dipoles of HeH^2+^. The black curves show the potential curves *C* of the first excited electronic state 2*pσ* and the second excited electronic state 2*sσ* of HeH^2+^. The red curves show the permanent dipoles *μ* of theses two states. The solid and dashed curves represent the 2*pσ* state and the 2*sσ*, respectively.

Generally, there are two physical effects that might lead to an energy shift of a bound state for an asymmetric molecule in an external laser field. The first one is the so-called Stark effect. The energy shift induced by the Stark effect can be given by^34^


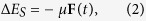


where *μ* is the permanent dipole of the electronic state, *F*(*t*) is the external field. In Fig. 4, we show the *R*-dependent permanent dipoles of the 2*pσ* (red solid curve) and 2*sσ* (red dashed curve) states^34^. Since *μ*_2*p*_(*R*) is positive, the energies of the 2*pσ* state synchronously oscillate with respect to -*F*(*t*). Moreover, because *μ*_2*p*_(*R*) increases with the increase of *R*, the amplitude of the oscillation of the 2*pσ* state also increases as the internuclear distance increases, as seen in Fig. 3. The 2*sσ* state exhibits more complex oscillatory behavior under the influence of the Stark effect. When *R* < 6 a.u., *μ*_2*s*_(*R*) is positive, leading to the similar oscillation with the 2*pσ* state. When *R* = 6 a.u., *μ*_2*s*_ is almost equal to zero. As a result, the energies of the 2*sσ* state almost undisturbed by the laser field. When *R* > 6 a.u., *μ*_2*s*_(*R*) becomes negative, resulting in an opposite oscillation with the 2*pσ* state.

The second effect that will lead to an energy shift of a bound state is the Autler-Townes effect (sometimes referred to as dynamical Stark effect)^35^,^36^. The Autler-Townes effect will lead to splitting of two degenerate molecular levels in a strong laser field. The separation for the two new eigenenergies of the “molecule + field” system caused by the Autler-Townes effect can be expressed as^36^


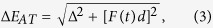


where Δ is the frequency detuning of the two molecular levels, *d* is the electric dipole transition moment of the two levels, *F*(*t*) is the instantaneous laser field. From equation (3), the Autler-Townes effect plays an important role only when Δ ≪ *F*(*t*)*d*.

With these two effects, we can explain the origin of the dip structure in Fig. 3(b) as well as the energy shift in Figs 1(d) and 2(a). For a small internuclear distance of *R* = 3.9 a.u. [see Fig. 3(a)], the unperturbed energy difference between the 2*pσ* and 2*sσ* states is large and the permanent dipoles of the 2*pσ* and 2*sσ* states are small. As a result, both Stark and Autler-Townes effects have a minor effect on the multiphoton above-threshold ionization of HeH^2+^. Therefore, there is no energy shift can be observed at small internuclear distances. For an intermediate internuclear distance of *R* = 6 a.u. [see Fig. 3(b)], *μ*_2*p*_ is large leading to a large oscillation of the 2*pσ* state due to the Stark effect. The Stark-shifted energy gap between the 2*pσ* and 2*sσ* states at the negative peaks of the laser field becomes much smaller compared to the unperturbed energy gap. Those two Stark-shifted states form a pair of quasi-degenerate states. The Autler-Townes effect becomes important, leading to the splitting of these two states. Thus a significant depression of the energies of the 2*pσ* state and a significant enhancement of the energies of the 2*sσ* state are observed. Therefore, one can see the obvious dips at the 2*pσ* state and sharp peaks at the 2*sσ* state at the negative peaks of the laser field. The dip structure increases the effective ionization potential of the electron at the first excited 2*pσ* state in the above-threshold multiphoton ionization process. Therefore, a clear energy shift can be observed in the JES of HeH^2+^ at this nuclear energy, as shown in Figs 1(d) and 2(a). In this case, both the Stark effect and the Autler-Townes effect play important roles. For a large internuclear distance of *R* = 9 a.u. [see Fig. 3(c)], the field-free molecular levels of the two states are quasi-degenerate, as seen in Fig. 4(see the black curves). In this case, as shown in Fig. 3(c), the 2*pσ* and 2*sσ* states show opposite oscillatory behavior influenced by the Stark effect. The oscillation amplitude of the 2*pσ* state is very large due to the large dipole of the 2*pσ* state. Therefore, the quasi-degenerate states at *R* = 9 a.u. become non-degenerate in the strong laser field because of the Stark effect. The Aulter-Townes effect becomes less important for HeH^2+^ at this internuclear distance. Thus the energy shift cannot be observed for the large internuclear distance (corresponding to nuclear energy of 0.22 a.u.) in the JES of HeH^2+^ molecule, as shown in Fig. 2(a,b). From the above analysis, one can see that the JES of the asymmetric molecules has carried the information of the *R*-dependent permanent dipole. The JES of Figs 1(d) and 2 depends on the permanent dipoles shown in Fig. 4. Thus the molecular structure might be retrieved from the JES in the future for more complex asymmetric molecules.

## Discussion

The energy shift in the JES of asymmetric HeH^2+^ is different from that of the symmetric 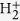
21. Because there is no permanent dipole for symmetric molecule, only the Aulter-Townes effect can play a role. Thus there is no energy shift for the low vibrational state of symmetric molecules. For the asymmetric molecule HeH^2+^, the energy shift of the electron-nuclear JES can be observed for *υ* = 0 vibrational state due to the strong Stark effect associated with nonzero permanent dipoles, as seen in Fig. 1(d). The Stark effect changes the energy gap between the states, leading to strong Aulter-Townes effect at a certain internuclear distance.

**Figure 5:**
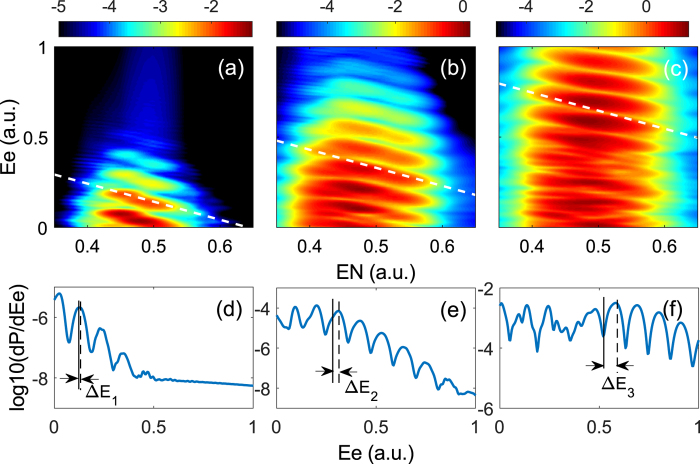
Electron-nuclear JES of HeH^2+^ (*υ* = 0) for three different laser intensities. (**a**) *I* = 7 × 10^13^ W/cm^2^, (**b**) *I* = 1.5 × 10^14^ W/cm^2^, and (**c**) *I* = 4 × 10^14^ W/cm^2^, respectively. The color scale is logarithmic. The dash lines indicate the absorptions of *n* = 13 photons (**a**), *n* = 15 photons (**b**), and *n* = 19 photons (**c**), respectively, in the processes. (**d**–**f**) show the cuts at the nuclear energies *E*_*N*_ = 0.5 a.u. for (**a**–**c**) respectively. The vertical solid lines and dashed lines indicate the positions of the peaks of the stripe and the positions of the white dashed line, respectively.

According to equation (2), the Stark shift is proportional to the laser electric field. Thus the Stark effect will become more important for higher laser intensity. One can expect that the electron-nuclei correlation might be controlled by varying the laser intensity for asymmetric molecules. In Fig. 5, we show the JES of HeH^2+^ molecule for different laser peak intensities. The laser intensities *I* = 7 × 10^13^ W/cm^2^ [Fig. 5(a)], *I* = 1.5 × 10^14^ W/cm^2^ [Fig. 5(b)], and *I* = 4 × 10^14^ W/cm^2^ [Fig. 5(c)], correspond to the Keldysh parameters 
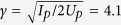
, 2.8, and 1.7, respectively (*I*_*p*_ is the ionization energy). Thus, at these intensities, above-threshold multiphoton ionization is dominant for the three cases. The slopes of these white dashed lines are −1. Generally, the stripes of the JES of HeH^2+^ gradually become horizontal as the laser intensity increases. One can see that the peak positions of the JES deviate from the dashed lines for higher laser intensities [Fig. 5(b,c)], while they are almost consistent with the dashed line at lower laser intensity [Fig. 5(a)]. To see this clearer, we show in Fig. 5(d–f) the cuts at the nuclear energies *E*_*N*_ = 0.5 a.u. corresponding to Fig. 5(a–c), respectively. The vertical solid lines and dashed lines in Fig. 5(d–f) indicate the positions of the peaks of the stripe and the positions of the white dashed line in Fig. 5(a–c), respectively. In Fig. 5(d), the difference between the dashed line and the solid line (Δ*E*_1_) is negligible. For higher intensities, the energy shift of the electron-nuclear JES becomes much more pronounced [Fig. 5(e,f)]. With the increase of the laser intensity, the energy shift increases, i.e., Δ*E*_3_ > Δ*E*_2_ > Δ*E*_1_. Consequently, the electron-nuclear energy sharing in the above-threshold multiphoton ionization of HeH^2+^ can be controlled by varying the laser intensity. Though our analysis is based on a one-electron molecule, the conclusion is not changed for two-electron and multielectron molecules with large permanent dipole moment. In a two-electron or multielectron molecule, both Stark effect and Autler-Townes effect can also play a significant role at certain laser condition depending on the potential energy curves. Thus the anomalous electron-nuclei energy sharing might be experimentally observed in two-electron and multi-electron molecules due to their higher stability.

In conclusion, we have investigated the correlated electron and nuclear dynamics for asymmetric molecule HeH^2+^. The electron-nuclear energy sharing of HeH^2+^ molecule subjected to a 400 nm laser pulse has been theoretically studied by solving the one-dimensional TDSE. We found an anomalous energy shift in the JES of HeH^2+^, inconsistent with the usual electron-nuclear energy sharing rule of the symmetric molecules. This energy shift can be observed at certain nuclear energies. Through tracing the time evolution of the electron wave packet of bound states in the laser pulse, we reveal that the bound-state energy oscillates with the laser fields due to the Stark effect associating with the permanent dipole of HeH^2+^. At certain internuclear distances, a pair of quasi-degenerate states (2*pσ* and 2*sσ* states) can be formed owing to the significant Stark shift of the states, leading to the strong Autler-Townes effect in the laser field. Only when both Stark effect and Autler-Townes effect play important roles, the energy shift in the JES of HeH^2+^ will appear. Because the Stark effect depends sensitively on the laser intensity, we further show that the electron-nuclear JES of HeH^2+^ is intensity-dependent. The stripes of the JES of HeH^2+^ become horizontal gradually as the laser intensity increases, meaning that the electrons share less energy with the nuclei at higher laser intensity. Therefore, by varying the laser intensity, we can control the electron-nuclear energy sharing in the above-threshold multiphoton ionization process of asymmetric molecules.

## Methods

### Reduced-dimensionality model

We numerically solve the TDSE of diatomic molecules (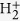, 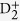, HD^+^, HeH^2+^) exposed in an linearly polarized laser field within the reduced-dimensionality model. The model consists of one-dimensional motion of the nuclei and one-dimensional motion of the electron. The model reproduces experimental results at least qualitatively^37^ and has been widely used to study the molecular fragmentation in strong fields^14^,^15^,^17^,^18^,^19^,^21^,^23^. We assumed that the electronic and nuclear motion are restricted along the polarization direction of the pulse which is parallel to the molecular axis. Then, the TDSE can be given as


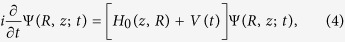


where *H*_0_ = *H*_*e*_(*z, R*) + *T*(*R*) is the field-free Hamiltonian with


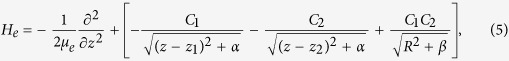


which includes the electronic kinetic energy and the electron-nuclear potential,


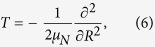


and *V*(*t*) is the electric potential with including the laser-molecule interaction. Here *R* is the internuclear distance and *z* is the electron coordinate with respect to the nuclear center of mass. *C*_1_ and *C*_2_ are the electric charges of the two nuclei. *z*_1_ = *m*_2_/(*m*_1_ + *m*_2_)*R* and *z*_2_ = −*m*_1_/(*m*_1_ + *m*_2_)*R* are the positions of the two nuclei, respectively. *μ*_*e*_ = (*m*_1_ + *m*_2_)/(*m*_1_ + *m*_2_ + 1) and *μ*_*N*_ = (1/*m*_1_ + 1/*m*_2_)^−1^ are the reduced masses with *m*_1_ and *m*_2_ as the masses of the two nuclei. For the symmetric diatomic molecules (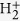, 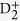, HD^+^), the soft-core parameters *α* = 1 a.u. and *β* = 0.03 a.u. are chosen so that the model yields the ground state energy of −0.7813 a.u. and equilibrium distance of 2.6 a.u., respectively. For asymmetric diatomic molecule (HeH^2+^), the soft-core parameters are chosen as *α* = 0.8 a.u. and *β* = 0.3 a.u. to yield the ground state energy of −0.8011 a.u. and equilibrium distance of 3.9 a.u., respectively. In the dipole approximation and the length gauge, the interaction with the laser field *F*(*t*) can be expressed by^33^


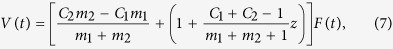


with *F*(*t*) = *F*_0_sin(*πt*/*τ*)^2^ sin(*ωt*). *F*_0_, *τ*, and *ω* are the peak electric field amplitude, pulse duration, and angular frequency, respectively. In the simulation, *ω* and *τ* are chosen as 0.114 a.u (*λ* = 400 nm) and nine optical cycles, respectively.

Before the time evolution of the wave function, an initial state is prepared. For the symmetric diatomic molecules, the ground state (electronic 1*sσ* state and *υ* = 0 state) is chosen to be the initial state of the system and is obtained by propagating the field-free Schrödinger equation in imaginary time^38^,^39^. For the asymmetric diatomic molecule, the lowest bound state is the first excited 2*pσ* electronic state. Thus in our calculation, the first excited state (electronic 2*pσ* state and *υ* = 0) is chosen to be the initial state of the system. The TDSE is solved on a grid using the Crank-Nicholson method with a time step of *δt* = 0.04 a.u.. We have used a box with |*z*| ≤ 2500 a.u. and *R* ≤ 25 a.u., with uniform grid spacings of *δz* = 0.2 and *δR* = 0.05. Then, the JES is obtained by using the method in ref. 16.

### Frozen nuclei model

In order to trace the time evolution of the electron wave packet of bound states, we further solve the TDSE of HeH^2+^ within the frozen nuclei approximation. In this model, the electronic TDSE can be written as


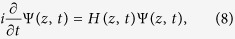


with the Hamiltonian


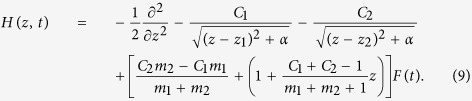


In the simulation, *z* ranges from −800 to 800 a.u. with 16000 points. The subscript 1 (2) represents to H^+^ (He^2+^). The soft-core parameter *α* is identical with that in equation (5). We introduce a mask function in the boundary to suppress the non-physical reflection from the simulation border.

Finally, the time evolution of the electron wave packet of bound states of HeH^2+^ can be calculated by applying the energy window operator^21^,^40^,^41^,^42^


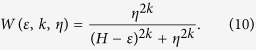


The time-dependent probability density of the energy *ε* can be obtained from


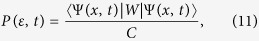


with 
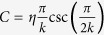
.

## Additional Information

**How to cite this article**: Wang, Z. *et al*. Correlated electron-nuclear dynamics in above-threshold multiphoton ionization of asymmetric molecule. *Sci. Rep.* **7**, 42585; doi: 10.1038/srep42585 (2017).

**Publisher's note:** Springer Nature remains neutral with regard to jurisdictional claims in published maps and institutional affiliations.
